# Ulceration and a White Lesion of the Tongue in a Male HIV Positive Patient: A Journey on the Avenue of Differential Diagnoses in Search of a Solution

**DOI:** 10.3390/life13040901

**Published:** 2023-03-28

**Authors:** Manuela Arbune, Monica-Daniela Padurariu-Covit, Elena Niculet, Iulia Chiscop, Anca-Adriana Arbune, Alin-Laurențiu Tatu

**Affiliations:** 1 Clinical Medical Department, Medicine and Pharmacy Faculty, “Dunarea de Jos” University of Galati, 800008 Galati, Romania; manuela.arbune@ugal.ro (M.A.); alin.tatu@ugal.ro (A.-L.T.); 2 Infectious Diseases Clinic Department I, Infectious Diseases Clinic Hospital “Sf. Cuv. Parascheva”, 800179 Galati, Romania; 3 Multidisciplinary Integrated Center of Dermatological Interface Research (MIC-DIR), “Dunarea de Jos” University, 800010 Galati, Romania; anca.arbune@icfundeni.ro; 4 The School for Doctoral Studies in Biomedical Sciences, “Dunarea de Jos” University of Galați, 800008 Galati, Romania; 5 Hematology Department, Emergency County Hospital “Sf. Apostol Andrei”, 800578 Galati, Romania; 6 Pathology Department, Emergency County Hospital “Sf. Apostol Andrei”, 800578 Galati, Romania; 7 Oral and Maxillofacial Surgery Department, Emergency County Hospital “Sf. Apostol Andrei”, 800578 Galati, Romania; iulia.chiscop@yahoo.com; 8 Clinical Surgery Department, Medicine and Pharmacy Faculty, “Dunarea de Jos” University of Galati, 800008 Galati, Romania; 9 Neurology Department, Fundeni Clinical Institute, 022328 Bucharest, Romania; 10 Dermato-Venerology Clinic Department, Infectious Diseases Clinic Hospital “Sf. Cuv. Parascheva”, 800179 Galati, Romania

**Keywords:** eosinophilic granuloma, HIV, tongue, white oral lesion, cannabinoids

## Abstract

Oral lesions are early indicator of immunosuppression, leading to HIV new diagnoses. The type of oral lesions can reveal opportunistic diseases that are correlated with the severity of immune depletion. Highly active antiretroviral therapy decreases the incidence of opportunistic oral infections, whereas a large variety of lesions are frequently experienced in people with HIV. Overlapping pathogenic mechanisms and multiple contributing etiologies are related to unusual, atypical oral lesions that are challenging in the clinical practice. We present a rare case of eosinophilic granuloma of the tongue in an older male HIV patient with severe immunosuppression due to the failure of antiretroviral treatment. Differential diagnoses considered squamous carcinoma, lymphoma, viral, fungal or bacterial infections and autoimmune disorders, as well as the influence of HIV immune disfunctions or the influence of cannabidiol use. The histopathologic and immunohistochemistry examination clarified the inflammatory reactive benign substrate of the lesion, although future survey of the oral lesions is essential.

## 1. Introduction

Almost 40 years since the identification of the human immunodeficiency virus (HIV) as the etiological agent of acquired immunodeficiency syndrome (AIDS), it is estimated that 84 million people have been infected and 38 million people are currently living with the virus. Despite the preventive measures supported by public health programs around the world, many new cases continue to occur, mainly sexually transmitted among men and in intravenous drug users [1].

The pathogenesis of HIV infection is chronic, progressive immunosuppression, initiated by the alteration of immune system cells, such as CD4 lymphocytes, macrophages, dendritic cells and mucosal cells, including cells of the oral mucosa [2].

Oral lesions are the earliest indicators of HIV infection, the expression of these lesions depends on the severity of the immunodepression [2,3]. It is estimated that 70–90% of people with HIV infection have oral lesions during their life. The increased susceptibility to oral lesions in the HIV population is a result of a combination of favorable factors, represented by systemic immunodeficiency through the depletion of CD4 lymphocytes, local immunodeficiency through the alteration of IL-17 producing cells, local conditions such as oral microbiota and dental biofilm or environmental factors such as smoking [3].

A large variety of oral lesions associated with HIV have been reported, but the highest prevalence is mentioned for oral candidiasis, oral hairy leukoplakia, herpes simplex virus infection (HSV), Kaposi’s sarcoma, non-specific ulceration, aphthous ulcers, periodontal disease, salivary gland disease, oral melanotic hyperpigmentation and oral warts. Next to oral manifestations attributed to immunodepression, people with HIV can present with independent lesions of a traumatic, autoimmune, neoplastic or malformity nature or as secondary reactions to some drugs or medications or the consequences of poor hygiene or dental abscesses, some of which may be intricate or simultaneous [4]. The use of antiretrovirals has changed the frequency and the presentation of the occurrence of oral lesions described in people with HIV. Patients with viral replication controlled by antiretroviral drugs have a lower prevalence of candidiasis, hairy leukoplakia of the tongue and oral Kaposi’s sarcoma but more frequently present papilloma and lesions of the salivary glands [2,5].

Oral lesions in people with HIV have a major impact on their quality of life, involving pain, difficulties in mastication and feeding, aesthetic damage, speech disorders and affecting social life. It is often difficult to diagnose these oral lesions because sometimes it can become interwoven, overlapping or self-sustaining. A correct diagnosis becomes important from the perspective of the therapeutic approach, both for basic conditions and for coexisting ones [6].

## 2. Case Presentation

A 64-year-old patient with acquired immunodeficiency syndrome (AIDS) was admitted to the our Infectious Diseases Department for the periodic evaluation of the immunosuppression syndrome. He complained of pain and paresthesia of the tongue and feeding difficulties.

The patient is currently pensioner, lives in an urban area and is divorced. He is an occasionally smoker, non-drinker and non-drugs user but has been taking cannabidiol-based supplements for the past three months.

He has family history of heart disease death (mother), prostate neoplasm (father) and intestinal occlusion (a brother).

From the personal history, we note severe and prolonged acute viral hepatitis 30 years ago, appendectomy, periodontitis, hypertension, stage G2 chronic kidney disease, external hemorrhoids, prostate adenoma and osteopenia. He was diagnosed with stage C3 AIDS (weight loss > 10%, oroesophageal candidiasis, persistent diarrhea) 7 years ago. He was evaluated for co-infections, indicating the absence of markers for hepatitis C, latent hepatitis B virus infection (hepatitis B s-antigen negative, hepatitis B core antibody negative, undetectable hepatitis B virus DNA) and serological markers for recent latent lues and was treated, according to guidelines, with benzathine penicillin [7].

Up to this point, he had received three types of antiretroviral combinations with lamivudine/abacavir/raltegravir, lamivudine/abacavir/zidovudine and emtricitabine/tenofovir/dolutegravir; all of which had therapeutic failure, although the resistance tests at the initiation of the last therapy revealed no resistance mutations and he was counseled for improving adherence (Table 1). After the HIV diagnosis, persistent candidiasis, episodes of pneumonia, anemia and leukopenia were reported. Additionally, he experienced a successful surgery for cataracts in his left eye. He also takes treatment for other chronic associated diseases including tamsulosin, indapamide and metoprolol. Drug–drug interactions with antiretroviral medication were not found.

**Figure 1:**
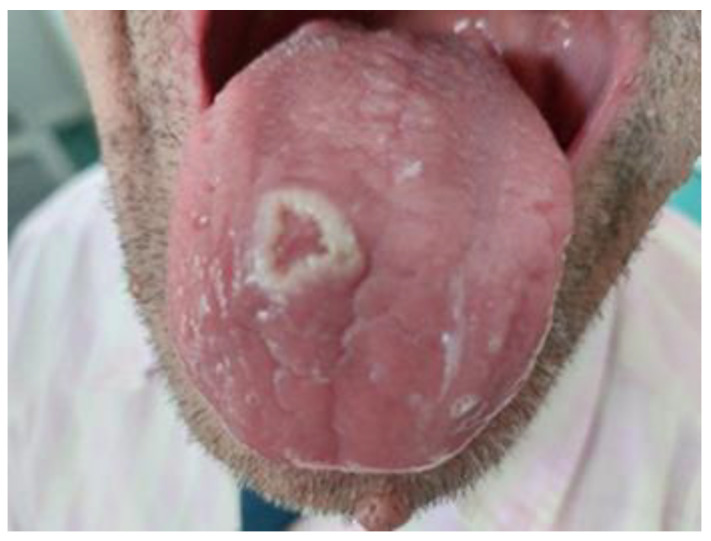
Tongue ulcer with overhanging margins (1st visit).

At the current medical visit, the patient was asthenic but cooperative and afebrile, with normal cardio-respiratory and neurological clinic parameters. The oral examination revealed dental prosthesis, deep fissures of the tongue and a painful well-defined circular crater-shaped lesion on the ventral side of the tongue with a diameter of about 8 cm and collar-like whitish, nonremovable, raised edges (Figure 1).

The otorhinolaryngology exam, posterior rhinoscopy, pharyngo–laryngoscopy and otoscopy were normal. Brain and chest contrast computed tomography did not reveal important pathological changes. The lab tests indicated CD_4_ lymphocytes below 50/mm^3^, persistence of HIV viral replication and hyposideremic anemia (Table 1).

**Table 1:** Yearly monitorization of the biological data on a male HIV-positive patient.

	2015	2016	2017	2018	2019	2020	2021	2022
Hb [g/dL]	12.3	14.3	14.5	14.6	11.8	13.7	13.8	11.4
Serum Iron	28.5	57.8	65.6	65.9	63.5	115.6	76.5	32.1
Platelets × 10^3^	202	196	188	242	217	237	208	184
WBC/mm^3^	2840	3600	5250	5790	3410	4700	5800	4800
Eos/mm^3^	154	266	168	92	51	169	116	130
Lymph/mm^3^	430	1170	1390	1140	760	1560	1410	1110
CD_4_/mm^3^	23	102	154	139	35	57	48	45
ARN-HIV c/mL	1,370,000	237	ND	270,707	10,482	20,166	109,497	233,000
VDRL	1/64	1/32	1/16	1/8	1/4	/	/	½
TPHA	++	++++	+++	+++	+	+	+	+

Legend: ND: undetectable; detection limit of HIV was 40 copies/mL.

The clinical aspect of the tongue lesion suggests an annular leukoplasiform lesion, taking into consideration the diagnoses of annular lichen planus, neoplasia and atypical lues but also of other white lesions of the tongue [8].

Ultrasonography examination was not available, though ultrasonography, and particular the use of high frequencies, could be a useful support for differential diagnoses [9]. 

**Figure 2:**
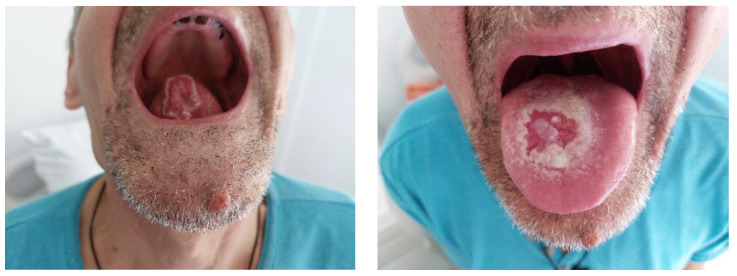
Ulceration in progress of epithelization after biopsy with perilesional leukoplaziform area (2nd visit, after 4 weeks).

A tongue biopsy was performed, and histopathological examination with standard hematoxylin–eosin staining revealed a fragment of lingual mucosa with ulceration, interstitial hemorrhages and reduced disseminated lymphohistiocytic inflammatory infiltrate. At the next visit, after 4 weeks, the oral examination revealed a white villous tongue with residual ulceration leukoplasia-like patches related to the epithelization process (Figure 2).

The lingual deposit fungal cultures revealed colonies of Candida albicans sensitive to flucytosine, amphotericin B, micafungin, voriconazole and fluconazole. The Xpert human papilloma virus (HPV) test was negative (HPV 16-18-45); however, the human herpes virus-8 (HHV8) IgG antibody and Epstein–Barr virus (EBV) IgG antibody tests were positive.

The patient continued antiretroviral therapy with emtricitabine/tenofovir/dolutegravir, pneumocystis prophylaxis with trimethoprim/sulfamethoxazole, antifungal treatment with fluconazole and the second standard dose of benzathine penicillin G for syphilis (total of three consecutive weekly administrations of 2.4 million units of intramuscular benzathine penicillin G).

**Figure 3:**
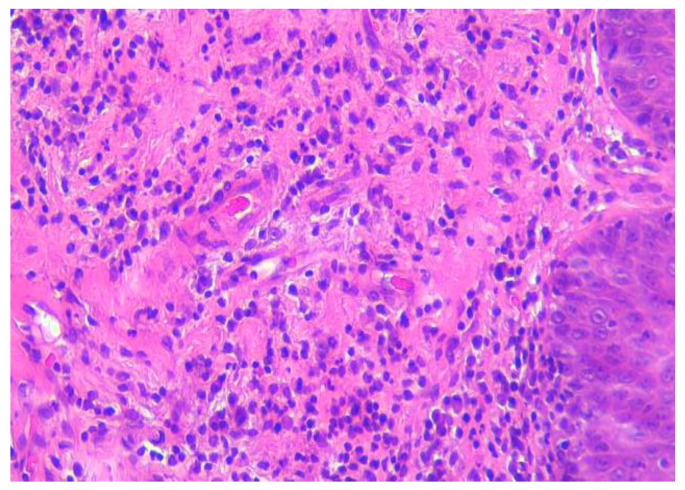
Hematoxylin and eosin (HE) staining of tongue biopsy tissue. There is abundant plasma cell infiltrate with lymphocytes and neutrophils immediately under the epithelium. 200×, HE.

We requested a second opinion for the histopathological sample, and it was described as a tongue fragment coated by non-keratinized squamous epithelium mucosa, with pseudoepitheliomatous hyperplasia and an area of ulceration with rich granulation tissue and polymorphous inflammatory infiltrates with numerous neutrophils, plasma cells, small lymphocytes, macrophages and eosinophils towards one of the extremities. Areas of diffuse, interstitial polymorphic inflammation are in the depth of the striated muscle tissue (Figure 3).

Periodic acid–Schiff (PAS) and Grocott’s methenamine silver (GMS) staining did not find fungal elements.

Immunohistochemistry (IHC) tests revealed a compatible aspect with ulcerated granuloma and eosinophils (CK5-6+ at the level of the covering epithelium; p40+; focal p53+, at the level of the covering epithelium; CD138+; CD20, CD79a+; CD3+; CD30+; P16; S100-; EBV-LMP1-; Ki67 proliferation index 5-10% at the level of the inflammatory infiltrate).

Both the histopathological aspect and the immunohistochemistry staining are compatible with the reactive nature of lymphoplasmacytic infiltrates [10,11,12].

**Figure 4:**
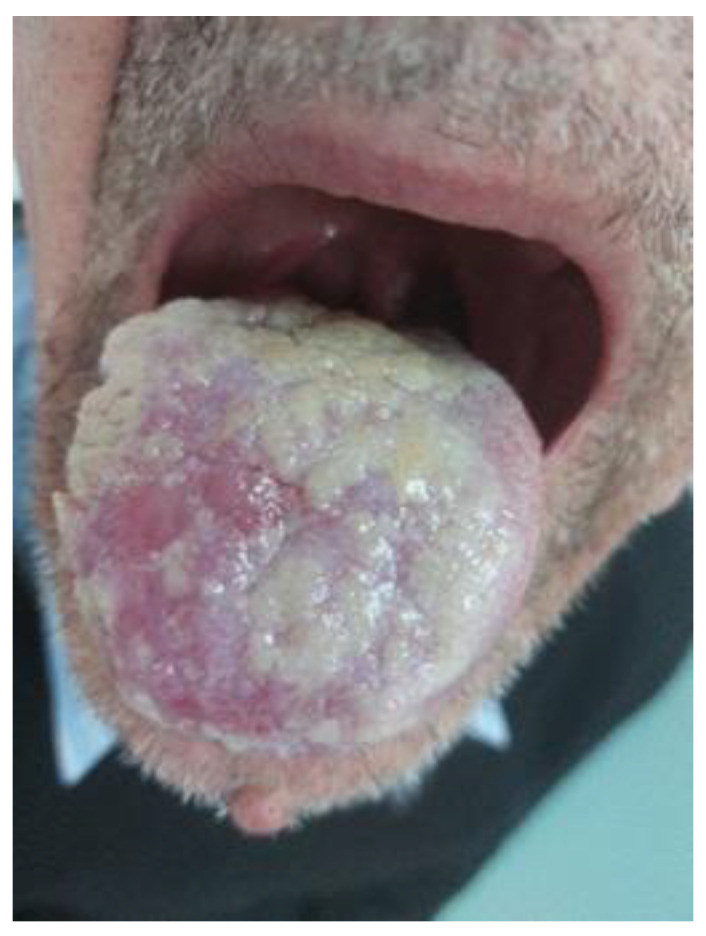
White, adherent lingual deposits; decreased lingual ulcer (3rd visit, after 8 weeks).

After 4 weeks, the oral examination described the epithelialization of the previously described ulcerated lesions, predominating the appearance of villous glossitis with abundant, irregular and unequal distribution of deposits, white, apparently creamy, thick unctuous-appearing lesions but adherent, on the anterior and dorsal surfaces of the tongue, including on the lingual frenum (Figure 4).

**Figure 5:**
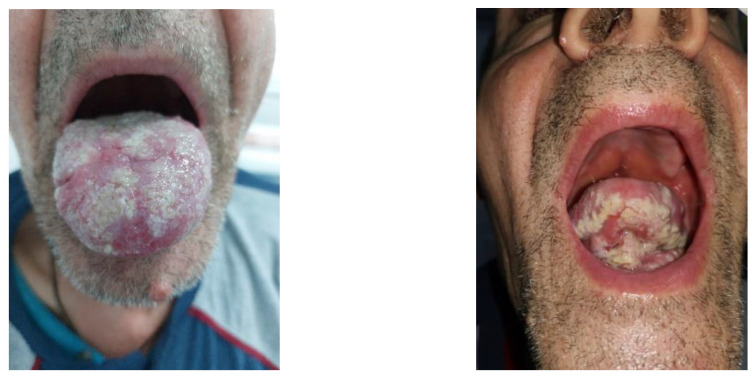
Shallow, round to oval depapillary erosions on a background of a whitish, non-wipeable hyperkeratotic thickening of the posterior aspect of the tongue in a pattern of plaques “en prairie fauchée” (the 4th visit, after 24 weeks).

The main treatment for the eosinophilic granuloma is surgical removal; however, other approaches are also described in the literature such as watch-and-wait, antibiotics, topical treatment, cryosurgery and intralesional or systemic corticosteroids [13]. The patient received a course of 3 weeks of antibiotic treatment for syphilis with benzathine penicillin G at 2,400,000 IU per week and mechanical removal of oral deposits. The oral discomfort was improved but the lesions’ appearance was slowly and incompletely regressed (Figure 5).

The clinical characteristic of the current patches corresponds to an “en prairie fauchee” pattern that was described in relation to syphilis, although other intricately etiologies could be involved in our case [14]. 

The final diagnoses for our patient are reactive inflammatory granulomatous leukoplastic glossitis, lingual candidiasis, late latent syphilis, severe acquired immunodepression syndrome and viral and immunological failure on antiretroviral therapy.

The management strategy for our patient includes the following interventions: careful oral hygiene and the systematic removal of deposits, treatment and primary prevention of the opportunistic infections and co-infections, monthly outcome report symptoms and oral clinical evaluation and adjustment of the antiretroviral regimen according to a new resistance test. Non-AIDS-related co-morbidities, such as blood hypertension, chronic kidney disease and osteopenia, should also be managed by a multidisciplinary team. The most important components of the management of this patient are to stop cannabidiol-based supplements, to identify unreported supplement use and to offer counseling and psychological support appropriate to the psychological stages of developing and maintaining adherence.

## 3. Discussion

The approach for oral HIV-related lesions is common in the medical practice, but atypical characteristics could be a serious challenge for clinicians.

### 3.1. Classification of Oral Lesions Related to HIV

Oral manifestations in HIV patients are classified into three groups according to the bond strength with the specific immunodepression to this viral infection (Table 2).

Fungal infections, oral hairy leukoplakia, Kaposi’s sarcoma, non-Hodgkin lymphoma and oral periodontal disease are the most relevant HIV-related clinical presentations.

### 3.2. Oral Eosinophilic Granuloma

Eosinophilic granuloma of the oral mucosa is a rare reactive lesion with a benign evolution. The pathogenesis is not yet clarified but an association with traumas has been observed in 50% of cases, contributing to ulcer development. Toxic or infectious agents can spread through the damaged tissues, and these agents are responsible for the inflammatory response stimulation. Eosinophil infiltrates are different from most traumatic ulcers, which are characterized by the presence of polymorphonuclear cells and the absence of eosinophils [16].

The clinical aspect is an isolated ulceration with indurated and elevated borders. Eosinophilic granuloma peaks in the fifth and sixth decade, with a minor predilection for occurring in men [13,17].

The lesions can spontaneously resolve after the removal of the supporting factors [18]. Different therapeutic approaches have been proposed: “watch and wait”, antibiotics and topical, intralesional or systemic approaches, as well as surgical interventions (curettage, cryosurgery, surgical excision) [16,19].

The present case corresponds to the demographic profile for traumatic ulcerative granuloma with stromal eosinophilia (sex, age), in which the traumatic factor may be the dental prosthesis. The ulceration decreased spontaneously, with the particularity of the development of secondary, white, villous lesions extended on both tongue surfaces.

The biopsy role in the lesion outcome could be controversial. Like the biopsied granuloma annulare, tissue repair could be expected and explained by hypothetical mechanisms such as cytokine syntheses by keratinocytes or Langerhans cells, attraction of inflammatory cells, induction of granulation tissue, extracellular matrix remodeling, vascular neogenesis or contraction of the wound [20]. Another hypothesis may be the reverse Koebner phenomenon present in certain dermatological conditions. Contrary to the Koebner phenomenon, the reverse Koebner response is the nonappearance or disappearance of the lesions of dermatoses at the site of a traumatic injury [21,22,23,24,25].

### 3.3. Syphilis and HIV Co-Infection

Syphilis is known as a great imitator of other dermatological diseases. HIV infection accelerates the evolution of syphilis by altering cell-mediated immunity and can be associated with atypical manifestations. In the secondary stage, along with the maculopapular and erythematous desquamative lesions, lichenoid, nodular or ulcerated lesions can rarely appear. Patients with HIV infection may have false-positive serological results even in the absence of reinfection and unusually high titers in nontreponemal tests, as well as prolonged false-positive or false-negative serological reactions. The existence of multiple plasma cells in the histopathological examination also raised the issue of lingual lesions in active syphilis [26].

The main lesions in the primary syphilis are the syphilitic chancres (primary syphiloma ulceration with an indurated base); in secondary syphilis, the lesions arise at the oral/lingual level are described as like mucous plaques, painful or asymptomatic, oval or crescentic and raised or thin erosions; tertiary syphilis is defined by goma with lingual ulcers [27,28].

However, a large variety of lesions are reported in the medical literature, such as erosive, bullous-erosive, pemphigus vulgaris, macular, papular or nodular, condyloma lata, leukoplakia-like, oral hairy leukoplakia-like or painless nodules on the tongue [27,28,29,30,31].

### 3.4. Differential Diagnoses of Oral White Lesions

The severe HIV immunodepression with long evolution and the failure of antiretroviral therapies, as well as latent coinfections, can contribute to the atypical, persistent and severe evolution of these lesions.

The differential diagnosis of white oral lesions, the clinical context and personal history exclude nicotinic stomatitis, uremic stomatitis and chemical burns [16]. Lichen planus is a T-cell-mediated autoimmune disorder that has been associated with HIV, HBV and atypical forms of lichen; however, the macroscopic appearance of the lesions in the presented case is different from the characteristics of the clinical forms of oral lichen, which are reticular, papular, atrophic/erosive, ulcerative or bullous [32].

Squamous cell carcinoma was invalidated by the histopathological and immunochemistry examination, which corroborated with the negative test for the main oncogenic types of HPV (16-18-45).

A hypertrophic candidiasis diagnosis could be considered according to the identification of fungus in the culture from the lingual lesions; however, these are adherent to the mucosa, suggesting the overlapping of candidiasis on another leukoplakia-type lesion. In addition, the standard treatment with fluconazole did not influence the aspect of the lesions.

A leukoplakia lesion looks like a white plaque that is homogeneous and adheres to the oral mucosa and cannot be classified in another entity. The etiology of these lesions is not known, but it may be supported by irritation caused by tobacco or local trauma.

Hairy leukoplakia is a particular form of leukoplakia, determined by the Epstein–Barr virus, with a folds or white ridges appearance that is usually located on the tongue lateral surfaces and is more common in people with immunodepression. In HIV infection, susceptibility to the development of hairy leukoplakia occurs from the stage of moderate immunodepression. Sometimes, the lesions can extend to the dorsal surface of the tongue and sublingual space and can combine with fungal lesions [33]. Knowledge of particular risk factors and clinical features of the lesions can help differentiate between candidiasis, leukoplasia and hairy leukoplakia (Table 3).

The patient has positive markers for previous infections with EBV and HHV8. Most primary EBV infections are asymptomatic, followed by carrier status. EBV has been associated with malignant conditions, for example Burkitt’s lymphoma, Hodgkin’s lymphoma, post-transplant lymphoproliferative disease and some gastric carcinomas [34].

Extranodal localization at the level of the head and neck non-Hodgkin’s lymphomas is 3–5%, and localization at the level of the tongue is extremely rare. The risk is higher in patients with rheumatoid arthritis and congenital or acquired immunodepression (AIDS) and those exposed to radiation or some drugs (phenytoin, digoxin, chemotherapy) [35].

EBV can express different oncogenic genes during latent infection, for example, latent membrane protein 1 (LMP1), which has shown oncogenic capacity in vivo and in vitro. Although in our case LMP1 was negative, this protein is detected by IHC and the classical method in only 20 and 60% of malignant tissue specimens, respectively. Even small amounts of LMP1, undetectable by classical IHC methods, can have the biological effects of activating NFkB signals that contribute to tumor cell growth [36].

HHV8 is a gamma herpesvirus with hematological oncogenic potential, such as primary effusion lymphoma (PEL), multicentric Castleman disease (MCD), germinotropic lymphoproliferative disorder (GLPD) and diffuse large B-cell lymphoma [30]. EBV and HHV8 co-infections constitute a challenge for diagnosis in the case of association with overlapping lesions and challenge oncological surveillance for the risk of developing a lymphoma [36,37,38].

### 3.5. Oral Lesions and Cannabidiol Use

The beginning of the oral lesions was at the same time as the start of the use of cannabidiol supplements. The way in which cannabinoids act on the lingual epithelium is not well known, but the expression of CB1 and CB2 receptors has been obvious in several immunohistochemistry studies. Moreover, the expression level of cannabinoid receptors influences the pathophysiological mechanisms of the tongue, especially in neoplastic and inflammatory processes. The safety of using these supplements is unknown, but a decrease in blood coagulability and interference with the liver enzyme system (changing the level of some drugs) have been reported [39].

The patient has a favorable attitude towards cannabinoids because it induces a relaxation state, relieves pain and stimulates the appetite; however, it also has negative effects—neuropsychic ones and ones in the oral cavity. The “dry mouth” sensation, the increased risk of dental caries and periodontal diseases and the inflammation associated with oral mucositis may be the consequence of the binding of cannabidiol metabolites to the receptors of the oral mucosa, the decrease of nitric oxide and the change in the oral microbiome [40,41].

## 4. Conclusions

The diagnosis of oral lesions in patients with HIV infections continues to be a challenge, even in the highly active antiretroviral era. Oral HIV-related pathology is related to HIV immunosuppression and has often overlapping mechanisms with various etiologies that are difficult to be recognized and interpretated. Eosinophilic granuloma of the tongue is a reactive inflammatory, benign lesion with human low incidence that should be systematically surveilled.

## Figures and Tables

**Table 2:** Classification of oral lesions related to HIV [15].

GROUP	Cause
GROUP I: Oral manifestations strongly associated with HIV	Fungal infections Candidiasis Erythematous Pseudomembranous Angular cheilitis
	Oral hairy leukoplakia
	Kaposi’s sarcoma
	Non-Hodgkin lymphoma
	Periodontal disease Linear gingival erythema Necrotizing gingivitis Necrotizing periodontitis Necrotizing stomatitis
GROUP II: Oral manifestations frequently associated with HIV	Bacterial infections Mycobacterium avium—intracellular Mycobacterium tuberculosis
	Viral infections Herpes simplex virus Human papilloma virus Condyloma acuminatum Focal epithelial hyperplasia Veruca vulgaris Varicella zoster virus
	Melanotic hyperpigmentation
	Salivary gland disease Xerostomia Swelling of salivary glands
	Ulcerations
GROUP III: Oral manifestations seen in HIV infections	Viral infections Cytomegalovirus Molluscum contagiosum virus
	Fungal infections Cryptococcus neoformans Histoplasma capsulatum Mucoraceae spp Aspergillus favus
	Bacterial infections Actinomyces israelii E. coli Kl. pneumoniae Bartonella henselae/quintana Syphilis
	Drug reactions

Adapted from: [15].

**Table 3:** Differential diagnosis of white lingual lesions in patients with HIV [16].

	Oral Leukoplakia	Oral Candidiasis	Oral Hairy Leukoplakia
Etiology	Unknown	Candida albicans	Epstein–Barr Virus
Risk factors	Tobacco, alcohol	HIV, diabetes, antibiotics, corticosteroids, radiotherapy, chemotherapy	HIV immunosuppression
Lesions	Plate, white, adherent	White, creamy plaques, easily removable, with underlying erythema	White, hyperkeratotic plaques with vertical striations aspect, adherent, located on the lateral surfaces of the tongue
Pain	No	Yes	No

Adapted from: [16].

## Data Availability

All data regarding the findings are available within the manuscript.
